# Eucaine Hydrochlorate, a New Local Anæsthetic

**Published:** 1896-09

**Authors:** Gaetano Vinci

**Affiliations:** Messina, Sicily


### EUCAINE HYDROCHLORATE, A NEW LOCAL ANÆSTHETIC.[1]

BY DR. GAETANO VINCI, MESSINA, SICILY.

Dr. Vinci has experimented with the preparation in the laboratory of Professor Liebreich, starting with the proposition that from its chemical composition it ought to show properties similar to those of cocaine. The new compound differs from cocalin in that a methyl group is substituted in it for a hydrogen atom which is formed by the action of ammonia upon acetone. The more convenient name of eucaine has been adopted in place of its chemical one of methyl-benzoyl-tetramethyl-gamma-oxypiperidine-carbonic acid methylester.

Like cocaine, its free base is with difficulty soluble in water, and forms large shining crystals, melting at 104° to 105° C. (220° to 222° F.); with acids it forms neutral salts having the same action as the base itself. The muriate of eucaine crystallizes from a watery solution in permanent shiny plates or scales, which contain one molecule of water of crystallization and have a formula of $C_{19}H_{27}NO_4HClH_2O$. They are soluble to the extent of six per cent. in water at the ordinary temperature. From methyl alcohol the hydrochlorate of eucaine crystallizes in shining prisms containing two molecules of methyl alcohol. Both forms of hydrochloric acid salt were investigated.

---

[1] Extract from a paper read at the session of the Hufeland'sche Gesellschaft, Berlin, April 16, 1896.

*Local Action.*—A two- to five-per-cent. solution of eucaine instilled into the eye of an animal, as a dog or rabbit, caused complete local anæsthesia in from one to three minutes. It began in the cornea, and spread from thence to the conjunctiva, and lasted on an average from ten to twenty minutes. It was readily prolonged by repeating the dose. It was always accompanied by a slight hyperæmia, and slight irritation of the palpebral conjunctiva. This was only the case with the methyl alcohol form; the watery solution caused at most a very slight hyperæmia. The pupil was not dilated, and reacted well to light. Injected under the skin eucaine caused complete anæsthesia of the part, so that the reflex could not be evoked even with a needle. A similar complete local anæsthesia of the mucosæ was affected when a eucaine solution was painted over it.

The general action of the drug, both in cold- and warm-blooded animals, consisted in a marked excitation of the entire central nervous system, followed by paralysis in toxic doses going on to death. Even two-thousandths gramme (one thirty-third grain) caused irritability, heightened reflexes, inco-ordination, and finally general paralysis in the animals experimented with. Small doses administered to mice and rabbits caused increased reflex excitability, and increased but weakened respiratory movements. Medium doses of two-hundredths to three-hundredths gramme (one-third to one-half grain) per kilogramme (thirty-five ounces) caused repeated tonic and clonic convulsions. The animals lay senseless on their sides, with dyspnœa, opisthotonos, and finally paresis of the posterior limbs. These phenomena were most marked when large toxic doses of ten-hundredths to fifteen-hundredths gramme (one and a half to two and a quarter grains) per kilogramme (thirty-five ounces) were administered; the convulsions returned continuously, and affected all the muscles of the body. The animals finally died when the paralysis reached the respiratory muscles.

When the dose was not a fatal one, the convulsions gradually ceased, the increased reflex excitability disappeared, and the paresis of the hind limbs slowly improved.

The effect of eucaine on the central nervous system is therefore at first excitant, and later, in toxic doses, paralyzing. The paralysis is a central one, for if the sciatic nerve of a frog poisoned with eucaine is exposed, and its peripheral end irritated with the induced current, the limb reacts in a normal manner.

As regards its action on the heart and the blood-vessels, the subcutaneous and intravenous injection of small and medium doses slows it on the average from twenty to thirty beats per minute, but without otherwise modifying the beats, or increasing the blood-pressure. This effect on the pulse is caused by the excitation of the central vagus; for section of the vagi causes an immediate increase of the pulse to the normal and above it, together with an increase of the blood-pressure. Death occurs from paralysis of the respiratory centres, for the heart continues to beat for some time thereafter.

In all these points eucaine is similar physiologically to cocaine. Yet there are some important differences which must not be forgotten. In the first place eucaine is *less poisonous than cocaine.* Whilst the animals treated with eucaine survived, other animals injected with the same doses of cocaine died. The pulse with eucaine is always decreased in frequency; with cocaine there is a primary acceleration. As regards their local action, the commencement of the anæsthesia, its duration and intensity, there is no difference between the two substances. But eucaine causes no ischæmia; on the contrary, vascular dilatation occurs. A further difference is that the pupils are not affected; mydriasis does not occur, and the reaction to light remains normal.

The clinical experimentation was done in Professor Schweigger's Ophthalmological Department of the University Clinic at Berlin, and on all the various maladies of the eye,—acute and chronic inflammation of the cornea and conjunctiva, dacryocystitis, operative procedures, removal of foreign bodies from the cornea, cauterizations, etc. Both preparations were employed in two-per-cent. solution and compared with similar cocaine applications. They showed that the two drugs were of like value in the human subject also, as regards the rapidity, duration, and intensity of the anæsthesia. This is complete, progresses from the cornea to the conjunctiva, appears from two to five minutes after the instillation, and lasts from ten to fifteen minutes. There is some hyperæmia, and a slight irritation of the palpebral conjunctivæ. Some patients complained of a slight transitory burning, but only when the methyl alcohol preparation was used. The watery solution caused no by-effects save a slight, hardly noticeable hyperæmia. It is, therefore, the solution to be preferred for practical use.

Another difference of great importance is that eucaine does not like cocaine, induce mydriasis and paralysis of accommodation. The pupil is not dilated at all, and reacts well to light; the accommodation remains normal.

This is a property of the greatest importance in practical ophthalmology, and favors the employment of eucaine in cases in which a production of ischæmia with the anæsthesia is not required. In violent inflammatory conditions of the eye, eucaine also promptly produces anæsthesia, but the ischæmic action fails, and consequently for such cases cocaine will have the preference. Both drugs diminish the intra-ocular pressure about equally.

Its last advantage is that *the eucaine solutions are permanent and do not, like those of cocaine, decompose when kept.* Cocaine solutions are decomposed when they are boiled for the purpose of sterilization, thereby losing their property as a local anæsthetic; and the decomposition products have an irritant effect if such a solution is employed. Solutions of eucaine on the other hand do not suffer decomposition even when boiled for a long time.

Eucaine has thus been shown by experimentation on animals and on the human subject, to have very marked local anæsthetic properties which render it worthy of being placed by the side of cocaine in ophthalmological practice. *It has the advantage over the latter in that it has no effect on the pupil or on accommodation; that it is less poisonous than is cocaine; and that, whilst the absence of ischæmic effects render it less suitable in certain cases, in others its slight hyperæmic action will be distinctly advantageous.*

Sanitätsrath Dr. Reichert, specialist for diseases of the nose, throat, and ears, at Berlin, has demonstrated the therapeutic value of eucaine in diseases of the throat and naso-pharynx. He has shown that not only does it cause *marked local anæsthesia, but that it is absolutely harmless in medicinal doses.* Of especial importance is the fact that he found that *it does not affect the heart,* which has often been found to be the case with cocaine, more especially in neurotic and weakly individuals. In diseases of the nasal mucosæ Dr. Reichert found that eucaine alone, without any other application, sufficed to effect a cure in many cases.

Dr. C. L. Schleich, of Berlin, certifies as follows:

For the purpose of comparing it with cocaine I employed eucaine on the mucous membrane in one to five-per-cent. solutions, and in my method of infiltration anæsthesia, in one to two per cent. solutions. *There is not the slightest doubt that it is just as efficacious as cocaine in causing anæsthesia of the mucous membranes.* It depresses the superficial sensitiveness to about the same degree; in fact it was somewhat stronger when used in the same doses. It does not cause the marked ischæmia that cocaine does. *I have not met with a single case of poisoning;* there was not even any alteration in the pulse frequency or tension. After using a dilute eucaine solution for the purpose of anæsthetic œdema in some twenty cases, it was very forcibly impressed upon me *that eucaine was less poisonous than cocaine.* Experiments made upon my own person showed that the anæsthesia of the wheals was complete, though the infiltration was not quite so painless as with cocaine. Judging by my experience, I am of opinion that for pure contact anæsthesia, as by painting the solution over the mucosæ, it should replace cocaine.

In dental practice we are able to report that Professor Warnekros, instructor of dentistry at the University of Berlin, has admitted that *eucaine is preferable to cocaine inasmuch as the anæsthesia is very prompt, and an increase in the rapidity of the cardiac pulsations has never been observed.—Deutsche medicinal Zeitung*, No. 34, April 27, 1896.

